# Adverse Childhood Experiences and Psychological Health in Patients with Myasthenia Gravis: A Study Incorporating an Online Positive Mental Health Learning Program

**DOI:** 10.3390/healthcare14040502

**Published:** 2026-02-15

**Authors:** Ming-Hsing Chang, Wen-Han Chang, Yu-Chan Li, Jiann-Horng Yeh

**Affiliations:** 1 Department of Neurology, Shin Kong Wu Ho-Su Memorial Hospital, Taipei 111045, Taiwan; neurotttttttt@gmail.com; 2 Taipei Branch Office, Teacher Chang Foundation, Taipei 104036, Taiwan; wenhang64@gmail.com; 3 Department of Thanatology and Health Counseling, National Taipei University of Nursing and Health Sciences, Taipei 112303, Taiwan; yuhchain@ntunhs.edu.tw; 4 School of Medicine, College of Medicine, Fu Jen Catholic University, New Taipei City 242062, Taiwan

**Keywords:** myasthenia gravis, adverse childhood experiences, psychological well-being, mental health BMI, online learning program, positive mental health

## Abstract

**Background/Objectives**: This study examined the prevalence of adverse childhood experiences (ACEs) among patients with myasthenia gravis (MG) and explored associations between ACE exposure and psychological outcomes. In addition, this study conducted a preliminary evaluation of an online “Positive Mental Health BMI Learning Program” and its association with changes in psychological well-being. **Methods**: A total of 77 patients with MG were included, with data collected between January 2024 and January 2025. Sociodemographic characteristics, ACE exposure, and psychological and disease-related indicators were assessed, including the Myasthenia Gravis Activities of Daily Living Scale (MG-ADL), the Myasthenia Gravis Quality of Life 15-item scale (MG-QOL15), the indicator of mental health BMI on well-being (mBMI), and the Patient Health Questionnaire-9 (PHQ-9). Using a single-group pre–post design, this exploratory pilot study examined associations between ACEs and psychological outcomes, along with pre–post changes among participants who completed the online program. **Results**: Among the 32 participants who completed the online program, mBMI scores showed an increase, primarily reflecting improvements in emotional stability (21.41 ± 4.70 to 23.03 ± 4.49, *p* < 0.01); however, in the absence of a control group, these changes cannot be attributed solely to the intervention. In contrast, no significant pre–post changes were observed in PHQ-9, MG-ADL, and MG-QOL15. Across the full sample, higher ACE exposure was associated with greater depressive symptom severity, as measured by the PHQ-9 (*p* < 0.05). Overall, 42.9% of participants reported at least one ACE, with emotional abuse being the most frequently endorsed, followed by parental separation or divorce and emotional neglect. **Conclusions**: ACE exposure was common among patients with MG and was associated with greater depressive symptoms. Participation in the online positive mental health BMI learning program was associated with improvements in positive psychological well-being.

## 1. Introduction

Between 1995 and 1997, the Centers for Disease Control and Prevention (CDC) and Kaiser Permanente in San Diego, California, collaboratively conducted a landmark study examining the long-term health effects of adverse childhood experiences (ACEs) [1]. This research involved more than 17,000 adult participants and examined the relationship between internal and external stressors experienced during childhood within the family of origin and health outcomes later in life, including the development of various physical and mental disorders. The findings consistently demonstrated a strong association between ACEs, including physical, emotional, and sexual abuse as well as household dysfunction, and a wide range of diseases later in adulthood. More than half of the participants reported at least one ACE, and over 25% reported two or more. Individuals with higher ACE exposure showed markedly elevated risks of obesity, alcoholism, substance use disorders, depression, suicide attempts, and multiple other health conditions. These findings suggest that ACEs represent an important risk factor influencing adult health. Since the publication of this foundational study, the impact of childhood adversity on physical and psychological well-being has gained widespread attention in both public health and clinical research. A large systematic review and meta-analysis of more than 250,000 adults across multiple populations showed that exposure to multiple ACEs was associated with consistently elevated risks across a wide range of adverse health consequences [2]. Individuals reporting four or more ACEs exhibited the highest risk levels. These adverse consequences included markedly increased risks of mental ill health, substance use, and interpersonal violence, as well as moderately elevated risks of chronic medical conditions such as cardiovascular and respiratory diseases.

More recently, Madigan et al. synthesized evidence from over 200 studies involving more than 500,000 adults and confirmed that ACEs are highly prevalent across populations and consistently associated with poorer physical and psychological health in adulthood. This large-scale meta-analysis further reinforces ACEs as a robust and pervasive early-life exposure associated with long-term physical and psychological health effects across diverse populations and settings [3]. Beyond population-based mental and physical health functioning, emerging evidence has also linked ACEs to autoimmune diseases in adulthood. Large cohort studies conducted in Iceland and the United Kingdom have demonstrated a dose–response association between cumulative ACE exposure and the prevalence of autoimmune conditions, including rheumatoid arthritis, systemic lupus erythematosus, Sjögren’s syndrome, and thyroid disease [4]. Importantly, a substantial proportion of this association was mediated through psychological distress, particularly symptoms of depression, anxiety, and post-traumatic stress. These findings suggest that ACEs may influence autoimmune disease risk partly through long-term psychological pathways.

In recent years, numerous studies in Taiwan have extensively examined ACEs. Studies have shown that ACE exposure is associated with disrupted family environments, adverse emotional development, and poorer health outcomes in adulthood, including worse self-rated physical health, more emotional or sleep disturbances, and a higher likelihood of previous suicidal ideation [5,6]. ACEs have been associated with psychological resilience including patients with chronic inflammatory conditions such as ankylosing spondylitis [7]. The study further concluded that ACEs may serve as a predictor of health status in this population, highlighting the potential role of early-life adversity in shaping long-term health outcomes across immune-mediated disorders.

Myasthenia gravis (MG) is an autoimmune disease with well-established physiological mechanisms and advanced treatment options. However, existing research on MG has largely focused on psychological adjustment after disease onset, and no studies to date have examined the relationship between MG and ACEs. To our knowledge, this is the first study to explore the potential association between MG and ACEs. Considering that approximately one-quarter of individuals with MG demonstrate suboptimal psychological well-being [8], and that a group intervention based on solution-focused brief therapy and self-efficacy theory has been shown to enhance psychological health in MG patients [9], we adapted this program into an online video-based format. The original intervention was developed from “the indicator of mental health BMI on well-being (mBMI),” which comprises three components: interpersonal support and intimacy (B), emotional stability and regulation (M), and self-worth and meaning (I). This model conceptualizes mental well-being as an integrated construct reflecting interpersonal, emotional, and self-related functioning. The online version was titled the “Positive Mental Health BMI Learning Program.” We hypothesized that this online intervention may provide benefits comparable to those of in-person group counseling, thereby strengthening patients’ coping abilities and supporting emotional stability and adjustment.

## 2. Materials and Methods

### 2.1. Study Objectives

The objective of this study was to examine the associations between ACEs and psychological outcomes in patients with MG, and to explore whether participation in an online “Positive Mental Health BMI Learning Program” was associated with changes in psychological well-being during treatment.

At the MG Center of Shin Kong Wu Ho-Su Memorial Hospital, previous research has primarily focused on physiological mechanisms and medical management, while nursing research has tended to emphasize biopsychosocial care during hospitalization. Far less attention has been directed toward patients’ psychosocial adaptation beyond the acute phase of illness. Therefore, this study focused on psychosocial factors related to MG within routine clinical care, with an emphasis on assessment of psychological well-being.

### 2.2. Research Procedures

#### 2.2.1. Study Design

This study employed a single-group, pre–post interventional design to evaluate the psychological effects of an online behavioral program in patients with MG. The intervention was non-randomized and involved no masking procedures. Accordingly, this study was designed as an exploratory, pilot investigation to examine preliminary associations among study variables, with the absence of a control group reflecting feasibility considerations. This design was chosen to address descriptive research questions regarding psychological well-being and ACE exposure in this clinical population.

#### 2.2.2. Conceptual Framework

This study was guided by a conceptual framework in which ACEs were hypothesized to be associated with psychological outcomes in adulthood, particularly depressive symptoms and positive psychological well-being. Sociodemographic characteristics (e.g., age, sex, income, and living arrangement) were considered background factors that may influence both ACE reporting and psychological outcomes. MG-related disease indicators were included to describe disease severity and disease-related quality of life. Participation in the online Positive Mental Health BMI Learning Program was conceptualized as a supportive exposure potentially associated with changes in positive psychological functioning. This framework was intended to guide variable selection and interpretation of associations.

#### 2.2.3. Intervention Framework and Integration of Mental Health Indicators

Building on the framework of the “Positive Mental Health BMI Group Counseling Program” [8], this study adopted an intervention approach grounded in solution-focused brief therapy and self-efficacy theory. The program was designed to enhance subjective well-being from a positive psychological health perspective, encouraging participants to take an active role in their own care by identifying existing coping strengths and expanding their application in everyday life contexts.

Solution-focused brief therapy is a strengths-based and goal-oriented approach that emphasizes identifying existing resources, recognizing prior successful experiences, and focusing on achievable future changes rather than elaborating on problems. In the present program, this orientation guided participants to reflect on past coping successes, clarify personal values, and formulate realistic self-management goals relevant to daily life with MG.

Self-efficacy theory highlights the role of perceived competence and confidence in managing emotional and behavioral challenges. Accordingly, the intervention design emphasized active participation, gradual skill consolidation, and reinforcement of individuals’ sense of agency through structured reflection, practice, and positive feedback.

These theoretical principles were operationalized within the framework of the original Positive Mental Health BMI Group Counseling Program, which conceptualizes psychological well-being across three core domains: interpersonal support and intimacy (B), emotional stability and regulation (M), and self-worth and meaning (I). Across the online program units in this study, intervention components and guided exercises were designed to strengthen these domains through solution-focused processes and self-efficacy–oriented strategies, including goal setting, identification of prior successes, and commitment to small, attainable actions.

To ensure conceptual coherence between the intervention framework and outcome evaluation, this study adopted the dual-indicator mental health framework proposed by Li et al., incorporating both the mBMI and the Patient Health Questionnaire-9 (PHQ-9) [10]. Within this framework, the mBMI serves as a positive mental health indicator capturing interpersonal, emotional, and self-related well-being, whereas the PHQ-9 provides a complementary assessment of depressive symptom severity. These two indicators were selected to comprehensively evaluate psychological outcomes associated with participation in the “Positive Mental Health BMI Learning Program,” which was delivered as an online video-based intervention.

#### 2.2.4. Recruitment and Intervention Delivery

Participants were recruited from the neurology outpatient clinic of Shin Kong Wu Ho-Su Memorial Hospital between January 2024 and January 2025, as described in Section 2.4. Eligible patients attending routine outpatient visits were consecutively invited to participate. After providing informed consent in person during outpatient visits, they completed baseline demographic information and pre-test questionnaires online. They were then invited to join a private Facebook community created for the intervention, which was used as the sole platform for accessing intervention materials and program participation.

Beginning in the month after recruitment closed, pre-recorded instructional videos were released monthly on the second Monday at 10:00 AM and delivered sequentially over a four-month period. All intervention materials were uploaded and managed by the research team according to a standardized delivery schedule. After completion of the final unit, participants were invited to complete all post-test questionnaires online.

### 2.3. Design of the Video Materials for the Positive Mental Health BMI Learning Program

To enhance accessibility and accommodate patients’ physical limitations, the original group-based intervention was adapted into a structured online video-based learning program. The “Positive Mental Health BMI Learning Program” consisted of pre-recorded lecture-style sessions delivered by a licensed counseling psychologist with expertise in psychological health promotion.

Each session was presented in an online seminar format using a dual-screen layout. The main screen displayed presentation slides, typically comprising approximately 20 slides per session, while a smaller inset window showed the speaker. Each unit lasted approximately 15 min and focused on one of the three core mBMI domains or their integration. The curriculum was structured into four thematic units:

1. Unit One: Introduction to Mental Health
  This unit explained the importance of mental health self-management and introduced the concept of mental health within a dual-indicator framework comprising both positive and negative dimensions. The unit introduced concepts related to mental health self-management and included guided self-assessment activities across three domains: interpersonal support, emotional management, and personal meaning and value.

2. Unit Two: Interpersonal Support and Intimacy
  This unit highlighted the importance of interpersonal relationships and strategies for their maintenance. It addressed common patient mindsets, such as feelings of inferiority and perfectionistic thinking, and offered practical adjustment strategies and skills for sustaining healthy interpersonal connections.

3. Unit Three: Emotional Stability and Regulation
  This unit focused on awareness, identification, and acceptance of anxiety-related emotional states commonly reported by patients. It introduced techniques that use the five senses to anchor participants in the present moment, encouraged mindfulness practices, and provided methods to enhance emotional stability and self-management.

4. Unit Four: Sense of Self-Worth and Meaning
  This unit emphasized living with passion and inner strength. Participants were guided to reflect on their illness and treatment experiences, recognize their personal strengths, and identify sources of meaning in life, thereby fostering a sense of greater self-acceptance. The unit also provided support in developing practical strategies to affirm their sense of self-worth.

The content incorporated common psychological scenarios encountered by patients with chronic illness and emphasized practical reflection and skill application.

After each monthly video release, a brief live online question-and-answer session was conducted to allow participants to seek clarification. In addition, participants were able to replay the recorded videos at any time and post questions within the private Facebook community. Questions were addressed by the presenting psychologist and a neurologist specializing in MG, providing both psychological and disease-specific perspectives.

### 2.4. Research Sample

This study was approved by the Institutional Review Board of Shin Kong Wu Ho-Su Memorial Hospital (IRB No. 20230731R). The research period was from 10 January 2024, to 9 January 2025. Participants aged 18 to 80 years were recruited during their medical visits to Shin Kong Wu Ho-Su Memorial Hospital.

#### 2.4.1. Inclusion Criteria

1. Patients with a confirmed diagnosis of MG based on acetylcholine receptor antibody testing, single-fiber electromyography, repetitive nerve stimulation, or chest computed tomography. No restriction was applied regarding time since MG diagnosis.
2. Individuals able to clearly express their own intentions.
3. Individuals without cognitive impairment or psychiatric disorders. (Cognitive status and psychiatric history were assessed based on participant self-report, routine clinical evaluation during outpatient visits, and review of medical records.)
4. Individuals who are able to use Facebook, agree to participate in the study, and provide written informed consent in person at the outpatient clinic.

#### 2.4.2. Exclusion Criteria

1. Individuals who do not have a diagnosis of MG.
2. Patients who are unable to communicate adequately in Mandarin, Taiwanese, English, or written form.
3. Individuals with head trauma, psychiatric symptoms, or cognitive impairment that may interfere with verbal expression.

### 2.5. Research Instruments

To describe the sociodemographic characteristics of the participants, eight background variables were collected: gender, age, educational level, marital status, living arrangements, participation in social groups, employment status, and monthly income. Participants provided responses based on their actual circumstances. This study used the ACE Scale to assess participants’ exposure to childhood adversity. In addition, pre- and post-test scores from four psychological and disease-related questionnaires were analyzed to examine changes in mental health following completion of the online “Positive Mental Health BMI Learning Program.” The four questionnaires were:

1. Myasthenia Gravis Activities of Daily Living Scale (MG-ADL): Assesses the functional impact of MG on daily activities across ocular, bulbar, respiratory, and limb-related domains.
2. Myasthenia Gravis Quality of Life 15-item (MG-QOL15): Measures quality of life in patients with MG.
3. The indicator of mental health BMI on well-being Questionnaire (mBMI): Evaluates psychological well-being across three positive mental health domains: interpersonal support (B), emotional stability (M), and sense of meaning and value (I).
4. Patient Health Questionnaire-9 (PHQ-9): Screens for depressive symptoms and grades their severity.

Each instrument captures a distinct dimension of health or psychological functioning, allowing for a comprehensive assessment of changes following the intervention. Validated Chinese versions of all instruments were used in this study.

#### 2.5.1. Adverse Childhood Experiences Scale (ACE Scale)

The ACE Scale was originally developed by Felitti et al. and is widely used to assess ACEs. As described in subsequent literature, including *The Deepest Well: Healing the Long-Term Effects of Childhood Adversity* by Dr. Nadine Burke Harris, the scale categorizes ACEs into ten types [1,11]:

1. Emotional abuse
2. Physical abuse
3. Sexual abuse
4. Emotional neglect
5. Physical neglect
6. Parental divorce or separation
7. Household member treated violently
8. Alcohol and/or drug abuser in the household
9. Someone chronically depressed, mentally ill, institutionalized or suicidal
10. Incarcerated household member.

The scale consists of ten yes/no questions assessing experiences before the age of 18. Each “yes” response is scored as one point, yielding a total score ranging from 0 to 10.

#### 2.5.2. Myasthenia Gravis Activities of Daily Living Scale (MG-ADL)

The MG-ADL scale was developed by Wolfe et al. as a modification of the Quantitative Myasthenia Gravis (QMG) Score [12]. It assesses the impact of MG on daily physical activities. The scale consists of 8 items measuring four different functional areas:

1. Ocular function (2 items): double vision, ptosis
2. Oropharyngeal function (3 items): speech, chewing, swallowing
3. Respiratory function (1 item): breathing
4. Limb muscle function (2 items): personal hygiene ability, mobility (e.g., transferring)

Each item is rated on a 4-point scale (0–3), with higher scores indicating greater impairment. The total score ranges from 0 to 24, with higher scores indicating greater disability. The MG-ADL has demonstrated a moderate correlation with the QMG score (Pearson’s r = 0.583, *p* < 0.001), supporting its concurrent validity [12]. This scale is widely used as a validated measure of functional disability in patients with MG.

#### 2.5.3. Myasthenia Gravis Quality of Life 15-Item (MG-QOL15)

The Myasthenia Gravis Quality of Life scale (MG-QOL) was developed by Mullins et al. as a disease-specific quality-of-life measure for MG patients [13]. It was adapted from the Multiple Sclerosis Quality of Life Scale, including 100 questions covering four dimensions: physical, social, emotional, and functional issues. Following item reduction through redundancy analysis, relevance assessment, and prioritization based on patient and physician feedback, the scale was shortened to 60 items organized into seven domains:

1. Mobility (9 items)
2. Symptoms (8 items)
3. Emotional Well-Being (11 items)
4. General Contentment (7 items)
5. Thinking and Fatigue (4 items)
6. Family and Social Well-Being (9 items)
7. Additional Concerns (12 items)

Further refinement was conducted with feedback from 15 MG patients, who assessed its clarity and relevance. Responses are rated on a 5-point Likert scale: 0 (Not at all), 1 (A little bit), 2 (Somewhat), 3 (Quite a bit), 4 (Very much). The MG-QOL demonstrated high internal consistency reliability, construct validity, and convergent validity [13].

Subsequently, Burns et al. developed a shorter, 15-item version (MG-QOL15) [14]. The 15 items were selected from four categories of the original 60-item MG-QOL:

1. Mobility (9 items)
2. Symptoms (3 items)
3. General Contentment (1 items)
4. Emotional Well-Being (2 items)

Higher MG-QOL15 scores indicate a greater negative impact of the disease on the patient’s quality of life. The scale has a Cronbach’s alpha reliability coefficient of 0.89, demonstrating strong internal consistency [14].

#### 2.5.4. The Indicator of Mental Health BMI on Well-Being (mBMI)

The mBMI was developed by Li Yu-Chan and is grounded in three core positive psychological skill components:

1. Befriend (B), representing interpersonal support and closeness
2. Mindfulness (M), representing emotional stability and awareness
3. Identity (I), representing self-worth and meaning.

The mBMI questionnaire consists of three self-rated items, one for each component, and serves as a measure of positive psychological well-being. Responses are rated on a 11-point numeric rating scale ranging from 0 to 10, with higher scores indicating greater positive psychological well-being overall and within each subdomain. A total mBMI score above 20.5 has been suggested as an indicator of good mental health [15].

Research has shown that both total mBMI scores and the individual Befriend (B), Mindfulness (M), and Identity (I) component scores are effective predictors of mental health outcomes, including short-form mental health continuum scores as well as social, emotional, and psychological well-being [16]. A validation study has demonstrated acceptable reliability and construct validity of the mBMI, supporting its use as a brief measure of positive psychological well-being. Specifically, higher scores in the Befriend (B) dimension are associated with greater social well-being, higher Mindfulness (M) scores are related to enhanced emotional stability, and higher Identity (I) scores correspond to greater psychological well-being [15]. These findings suggest that the mBMI is a simple and effective instrument for assessing positive psychological well-being. The brief three-item structure was considered appropriate for this exploratory pilot study, as it captures key domains of positive mental health while minimizing participant burden in an online intervention context.

#### 2.5.5. Patient Health Questionnaire-9 (PHQ-9)

The PHQ-9, developed by Kroenke et al., is a self-report questionnaire designed to assess the frequency of depressive symptoms over the past two weeks [17]. Higher total scores reflect more severe depression. The scores are categorized into five levels of depression severity:

1. Minimal (0–4 points)
2. Mild (5–9 points)
3. Moderate (10–14 points)
4. Moderately severe (15–19 points)
5. Severe (20–27 points)

As the total score increases, the predictive value and likelihood ratio for major depressive disorder also rises, supporting the scale’s clinical utility in screening and severity assessment.

This study adopts the Chinese version of the PHQ-9 validated by Liu et al., which was tested in a sample of 1954 patients [18]. The results demonstrated good psychometric properties, with an internal consistency (Cronbach’s α) of 0.80 and a test–retest reliability of 0.87. Using a cutoff score of ≥10, the sensitivity for diagnosing major depressive disorder according to Diagnostic and Statistical Manual of Mental Disorders-IV (DSM-IV) criteria was 0.86, and the specificity was 0.94.

### 2.6. Safety Monitoring

Because this study involved minimal-risk behavioral intervention, participants were monitored for any potential adverse events throughout the intervention period.

### 2.7. Data Analysis

All statistical analyses were performed using IBM SPSS Statistics for Windows, version 22.0 (IBM Corp., Armonk, NY, USA). Given the exploratory nature of the study and the sample size of this study, analyses were primarily descriptive. Group comparisons and pre–post analyses were selected to characterize patterns of association rather than to formally test causal hypotheses. No formal a priori power analysis was conducted because the study was designed as an exploratory and hypothesis-generating investigation.

Descriptive statistics, including frequencies, percentages, means, and standard deviations, were used to summarize participants’ sociodemographic characteristics and study variables. Paired-samples *t*-tests were used to describe pre–post differences in psychological and disease-related measures among participants who completed the intervention. One-way analysis of variance (ANOVA) was used to examine differences across ACE score groups. When appropriate, Scheffé or least significant difference (LSD) post hoc tests were applied based on the homogeneity of variances as assessed by Levene’s test. A two-tailed *p*-value of <0.05 was considered statistically significant.

Item-level missing data were minimal because questionnaires were completed electronically with required response fields. Analyses were conducted using available complete data, and no imputation procedures were applied.

## 3. Results

**Figure 1:**
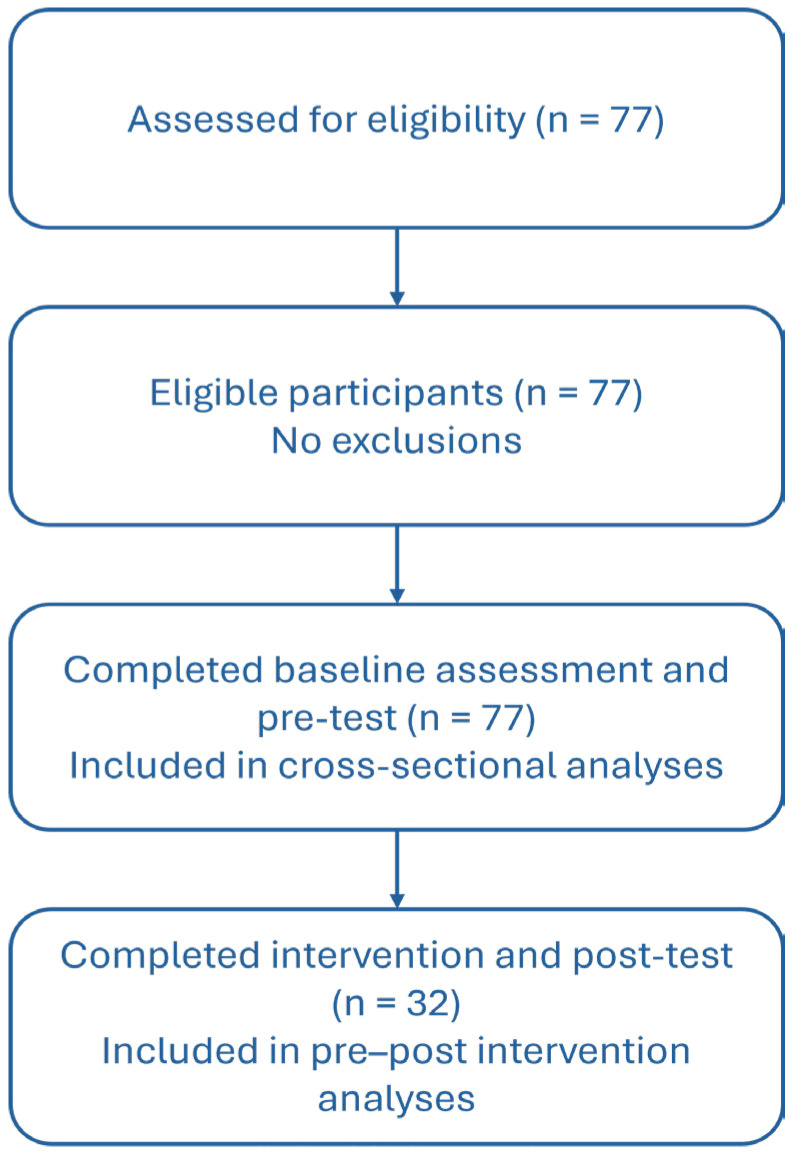
Flowchart of participant enrollment, intervention completion, and data analysis.

No participants were excluded after eligibility assessment. A total of 77 participants completed baseline assessments and the online pre-test questionnaire, and were included in cross-sectional analyses. Pre–post intervention analyses were restricted to the 32 participants who completed both the intervention and post-test assessments (Figure 1).

### 3.1. Sociodemographic Description of the Sample Group

Table 1 summarizes the sociodemographic characteristics of the 77 study participants, including 59 females (76.6%) and 18 males (23.4%). The largest age group was middle-aged adults (45–64 years; *n* = 41, 53.2%), followed by young adults (18–44 years; *n* = 28, 36.4%) and older adults (≥65 years; *n* = 8, 10.4%). In terms of education, 64.9% of participants (*n* = 50) had attained a college degree or higher, and 58.4% (*n* = 45) were married. The majority lived with family members (*n* = 69, 89.6%), and 61 participants (79.2%) reported participation in community groups. Regarding employment, 54.5% (*n* = 42) were employed full-time. With respect to income, 53.2% of participants (*n* = 41) reported a monthly income of NT$30,000 or more.

### 3.2. Endorsement Rates for ACE Questionnaire Items

In the ACE questionnaire (Table 2), the most frequently reported experience was emotional abuse (*n* = 15, 19.5%), followed by parental divorce or separation (*n* = 13, 16.9%) and emotional neglect (*n* = 11, 14.3%). The least frequently reported experiences were having a household member who was treated violently (*n* = 2, 2.6%) and having an incarcerated household member (*n* = 2, 2.6%), followed by physical neglect (*n* = 3, 3.9%). The remaining four ACE categories showed intermediate prevalence rates, including alcohol and/or drug abuse in the household (*n* = 5, 6.5%), physical abuse (*n* = 9, 11.7%), sexual abuse (*n* = 7, 9.1%), and having a household member who was chronically depressed, mentally ill, institutionalized, or suicidal (*n* = 7, 9.1%).

### 3.3. Sociodemographic Factors and ACEs

As shown in Table 3, analyses indicated that the total ACE score differed across age groups (*p* = 0.047) and living arrangement categories (*p* = 0.002). At the item level, age was associated with differences in endorsement of emotional abuse (*p* = 0.020), physical abuse (*p* = 0.024), and sexual abuse (*p* = 0.008). Living arrangement was associated with differences in several ACE items, including sexual abuse (*p* = 0.004), emotional neglect (*p* = 0.048), household member treated violently (*p* < 0.001), and having a household member who was chronically depressed, mentally ill, institutionalized, or suicidal (*p* = 0.004). Monthly income was not associated with the total ACE score; however, differences in emotional abuse endorsement across income categories were observed (*p =* 0.035).

#### 3.3.1. Age and ACEs

Post hoc multiple comparisons indicated age-related differences in endorsement of ACE items 1–3 (Table 4). For emotional abuse, participants aged ≥ 65 years reported higher scores than those aged 45–64 years (*p* = 0.03). For physical abuse, participants aged 18–44 years reported higher scores than those aged 45–64 years (*p* = 0.015). Similarly, for sexual abuse, higher scores were observed among participants aged 18–44 years compared with those aged 45–64 years (*p* = 0.009).

#### 3.3.2. Living Arrangement and ACEs

Analyses indicated that ACE item endorsement differed across living arrangement categories for sexual abuse (*p* = 0.004), emotional neglect (*p* = 0.048), household member treated violently (*p* < 0.001), and having a household member who was chronically depressed, mentally ill, institutionalized, or suicidal (*p* = 0.004). Post hoc multiple comparisons were not performed because one living arrangement category (“other”) included only a single participant, resulting in insufficient cell sizes for reliable pairwise comparisons. Consequently, differences among specific living arrangement categories (e.g., living alone versus living with family) could not be examined.

#### 3.3.3. Monthly Income and ACEs

In Table 5, post hoc comparisons indicated higher emotional abuse scores reported by participants with no monthly income compared with those with high income (mean difference = 0.2898, *p* = 0.039). No significant differences were observed between the none and low income groups or between the low and high income groups.

### 3.4. Associations Between ACEs and Psychological/MG-Related Scales

Table 6 shows that more than half of the participants (*n* = 44, 57.1%) reported no ACEs. Among the remaining participants, 18.2% (*n* = 14) reported one ACE, 10.4% (*n* = 8) reported two ACEs, 6.5% (*n* = 5) reported three ACEs, and 7.8% (*n* = 6) reported four or more ACEs.

Across the entire sample, the MG-ADL had a mean score of 3.74 (Standard deviation, SD = 2.97), and the MG-QOL15 had a mean score of 12.61 (SD = 11.58). For psychological well-being, the mBMI questionnaire yielded an overall mean score of 22.36 (SD = 5.63). Regarding depressive symptoms, the PHQ-9 had a mean score of 6.16 (SD = 5.35).

ANOVA results indicated no significant differences in MG-ADL, MG-QOL15, or mBMI scores across ACE total score groups (Table 6). In contrast, PHQ-9 scores differed significantly across ACE total score groups (*F* = 2.931, *p* = 0.014), with a trend toward higher mean PHQ-9 scores among participants with greater ACE exposure.

### 3.5. Effects of the Online Positive Mental Health BMI Learning Program on Pre–Post Outcomes

Table 7 shows that the MG-ADL scale had a mean pre-test score of 4.00 (SD = 2.98) and a post-test score of 3.47 (SD = 2.86), with no significant difference between the two. Similarly, the MG-QOL15 yielded pre-test and post-test mean scores of 15.66 (SD = 13.26) and 14.06 (SD = 10.24), respectively, with no significant change. PHQ-9 scores decreased slightly from 6.25 (SD = 4.40) at pre-test to 5.94 (SD = 4.31) at post-test; however, this reduction was not statistically significant. As pre–post analyses were restricted to participants who completed the intervention, these individuals may represent a more motivated or higher-functioning subset of the original sample, which limits the generalizability of the observed pre–post findings.

In contrast, mBMI scores increased significantly from 21.41 (SD = 4.70) at pre-test to 23.03 (SD = 4.49) at post-test (*p* = 0.007), as shown in Table 7. The pre–post change in mBMI showed a medium within-subject effect (Cohen’s d_z_ ≈ 0.51), with a more conservative estimate based on averaged standard deviations (Cohen’s d_av_ ≈ 0.35). Using the suggested mBMI cut-off of 20.5, 19 of the 32 participants were classified as having “good mental health” at baseline, compared with 25 participants at post-test. This corresponded to 7 participants shifting from the “poor” to the “good” mental health category over the intervention period. One participant showed a shift from the “good” to the “poor” category. Subscale analyses (Table 8) indicated that this increase was primarily observed in the Mindfulness (emotional stability and regulation) component (Item 2: “How confident are you in detecting changes in your inner emotions and keeping yourself calm?”), which showed a statistically significant pre–post difference. In the absence of a comparison group, the observed changes should be interpreted descriptively and cannot be attributed solely to the intervention. They may reflect natural history, placebo effects, or repeated testing.

### 3.6. Adverse Events

No adverse events were observed or reported during the intervention period.

## 4. Discussion

### 4.1. Association Between MG and ACEs

The present study did not identify a high prevalence of ACEs within this outpatient MG cohort. As shown in Table 6, 57.1% of participants reported no ACEs, and only 7.8% reported four or more ACEs. This proportion is lower than that reported in large population-based studies, such as the CDC–Kaiser ACE Study, in which more than half of adults reported at least one ACE [1]. Differences in sampling strategies, cultural context, and clinical data collection may contribute to this discrepancy. As a result, reported ACE exposure in this clinical MG sample appeared relatively limited.

Despite the absence of a high overall ACE burden, greater ACE exposure was associated with increased depressive symptom severity, as reflected by higher PHQ-9 scores. This pattern is consistent with previous research indicating that adverse early-life environments may influence emotional development, stress responsivity, and vulnerability to depressive symptoms in adulthood. Prior study has linked ACEs to long-term psychological outcomes, including impaired emotional regulation, maladaptive coping behaviors, depression, suicidality, substance use, and metabolic disorders [5].

Evidence from other chronic medical conditions suggests that exposure to adverse experiences during childhood may be associated with psychological symptoms and reduced quality of life in adulthood. For example, studies in patients with recurrent aphthous stomatitis have reported higher levels of childhood trauma alongside increased depressive symptoms and psychological distress compared with controls, which were further associated with poorer quality of life [19]. These findings offer additional support for the observed associations between early-life adversity and psychological health in chronic illness.

The three most frequently reported ACEs in this study were emotional abuse, emotional neglect, and parental marital disruption. Previous research has consistently shown that these forms of early-life adversity are associated with adverse emotional and psychological outcomes, including challenges in emotional development, perceived security, and self-worth. Disruptions in family stability and caregiving environments during childhood have been linked to greater emotional vulnerability and difficulties in affect regulation later in life [1,5]. Such associations may be relevant for individuals living with chronic medical conditions, including MG, who often face ongoing illness-related stressors that can further challenge psychological well-being. Other ACE categories, including physical abuse, domestic violence, sexual abuse, and exposure to household mental illness or suicidality, were reported by approximately 10% of participants. Although these rates are lower than those reported in Western population-based samples such as the CDC–Kaiser ACE Study by Felitti et al. [1], these experiences remain established risk factors for subsequent psychological and behavioral difficulties. In contrast, ACEs involving household substance abuse, criminal behavior, or incarceration were relatively uncommon in this cohort, with prevalence rates generally below 5%. Cultural context, social norms, and potential underreporting in clinical settings may partly contribute to these observed differences.

Age-related variation in ACE endorsement was identified among Taiwanese patients with MG. Participants aged 65 years and older reported a higher prevalence of emotional abuse before the age of 18 compared with those aged 45–64 years, whereas participants aged 18–44 years reported higher rates of physical abuse than the middle-aged group. Childhood sexual abuse was also reported more frequently among younger adults than among participants aged 45–64 years.

These age-related differences may reflect cohort-related variation in the interpretation, recognition, and reporting of ACEs, as well as possible differences in actual exposure. Evolving conceptualizations of abuse, changes in sociocultural norms, and increasing public awareness of psychological well-being and trauma-related concepts over time may influence how childhood experiences are identified and disclosed. In addition, differences in recall and reporting practices across age groups cannot be excluded. Accordingly, retrospective reports of ACEs should be interpreted cautiously when comparing across age groups.

An income-related difference was detected for emotional abuse, with participants reporting no monthly income showing higher endorsement rates than those with higher income levels. Previous longitudinal and cross-sectional studies have shown that childhood exposure to abuse, neglect, and household dysfunction is associated with increased risks of unemployment, poverty, and lower occupational attainment in adulthood [20,21]. In the present study, no significant differences were noted between the none and low income groups or between the low and high income groups, suggesting that the observed association was limited to contrasts between the lowest and highest income categories. Given the small sample size and limited subgroup cell counts, this income-related finding, along with other subgroup analyses by ACE count or sociodemographic factors, should be interpreted as exploratory and considered hypothesis-generating only, requiring validation in larger cohorts.

### 4.2. Effects of the Online Positive Mental Health BMI Learning Program Intervention

The study employed a single-group pre–post design without a control condition; therefore, the observed changes in mBMI cannot be attributed to the intervention with causal certainty. Instead, the findings provide an initial indication of associations between participation in the online program and psychological outcomes among patients with MG.

Following completion of the “Positive Mental Health BMI Learning Program,” an increase in overall mBMI scores was noted, with the most evident improvement in the domain of emotional stability and regulation. In contrast, no statistically significant pre–post changes were found in MG-related outcomes (MG-ADL and MG-QOL15) or depressive symptoms as measured by the PHQ-9. This pattern suggests that participation in the program was more closely associated with changes in positive psychological functioning than with changes in disease-related indicators or depressive symptom severity.

The differential pattern observed across psychological outcomes may reflect fundamental differences between positive mental health constructs and symptom-based measures of depression. The mBMI focuses on positive psychological resources such as emotional stability, self-regulation, interpersonal support, and self-worth, which are directly targeted by the intervention content. In contrast, the PHQ-9 assesses the severity of depressive symptoms and may be less sensitive to short-term changes in positive psychological functioning in the absence of depression-specific treatment, consistent with dual-continua models of mental health and prior meta-analytic findings from positive psychology interventions [22,23].

Similar patterns have been reported in previous group-based interventions targeting subjective well-being among individuals with MG, in which improvements in mental health-related outcomes were observed without parallel changes in disease severity [9]. Recent syntheses of online mindfulness-based programs in populations with chronic health conditions have suggested benefits for psychological functioning, although the magnitude and domains of change vary across outcomes and study settings [24]. The same review also highlighted substantial heterogeneity in intervention components and limited tailoring to specific patient populations, underscoring the need for developing and evaluating tailored online psychological interventions for specific clinical populations.

Evidence from psychological interventions in other neuroimmune chronic conditions further supports this pattern of outcomes. A systematic review and meta-analysis of randomized controlled trials demonstrated that mindfulness-based interventions improve mental well-being in people with multiple sclerosis, including interventions delivered in both in-person and online formats [25]. Complementing these efficacy findings, qualitative syntheses indicate that online mindfulness-based interventions are acceptable and feasible for people with multiple sclerosis, particularly when programs are tailored to disease-related limitations and emphasize accessibility, shared experiences, and instructor support [26]. Consistent with these findings, a broader systematic review of Internet-based psychological interventions, including mindfulness-based and other psychological approaches, reported potential benefits for depressive symptoms, anxiety, stress, fatigue, and quality of life in this population, while also noting heterogeneity across outcomes and study designs [27]. Beyond neuroimmune conditions, internet-delivered positive psychology interventions in other chronic conditions, such as chronic pain, indicate that improvements in psychological well-being are achievable even when reductions in clinical symptoms are modest [28].

From a broader psychological perspective, integrative models of mental health emphasize the capacity to integrate emotional experiences, self-perception, and adaptive regulation across changing life circumstances. These models highlight the importance of emotional awareness, regulation, and self-related coherence in maintaining psychological well-being, particularly following adverse or stressful experiences. Interventions that strengthen emotional stability and self-regulatory capacity may be especially relevant for individuals with chronic illness, who must continuously adapt to both disease-related and psychosocial stressors [29].

The online format offers practical advantages for this population, including flexibility, accessibility, and reduced physical burden, which may help lower barriers to participation during ongoing disease management. In this context, an online psychological program may serve as a supportive component of comprehensive care aimed at promoting psychological resilience.

### 4.3. Limitations

Several limitations should be considered when interpreting the findings of this study. First, the intervention was evaluated using a non-randomized, single-group pre–post design without a control or comparison condition. Therefore, causal inferences regarding intervention effects cannot be drawn, and observed changes in psychological outcomes may also reflect regression to the mean, expectancy effects, or external influences.

Second, attrition occurred over the course of the intervention, with 32 of the 77 enrolled participants completing the post-test assessments. Participation in the online program was voluntary, and adherence to all intervention components was not formally enforced or systematically monitored. Reasons for non-completion were not collected in a structured manner. Consequently, baseline characteristics were not formally compared between completers and non-completers. Potential systematic differences between completers and non-completers, including motivation, baseline psychological status, digital accessibility, or disease stability, cannot be excluded. The intervention-related findings should therefore be interpreted as reflecting outcomes among program completers.

Third, the duration of follow-up was relatively short and limited to immediate post-intervention assessment. As a result, the persistence and long-term stability of the observed psychological changes could not be evaluated.

Fourth, several subgroup and post hoc analyses were conducted based on small cell sizes. These analyses should be interpreted cautiously, as the limited sample size reduces statistical stability and constrains the strength of subgroup-level inferences.

Fifth, multiple statistical tests were performed across ACE domains, demographic variables, and psychological outcomes. Considering the exploratory nature of the analyses, no formal correction for multiple comparisons was applied, which may increase the risk of Type I error. Accordingly, statistically significant findings should be interpreted cautiously, particularly those from subgroup and secondary analyses.

Sixth, residual confounding cannot be excluded. Unmeasured or incompletely measured factors, such as illness duration, treatment intensity, baseline psychological characteristics, social support, or concurrent life stressors, may have influenced both ACE reporting and psychological outcomes, as well as participation and response to the online program. These unmeasured factors could partly account for the observed associations, and the direction and magnitude of such confounding effects cannot be determined within the present exploratory design.

Finally, ACE exposure was assessed using retrospective self-report measures, which are subject to recall bias and potential cohort-specific reporting differences. Measurement invariance across age groups was not formally examined, which should be considered when interpreting age-related patterns. In addition, the study sample was drawn from a single medical center in Taiwan, and participation in the intervention required access to and familiarity with a social media platform. Cultural context, patterns of technology use, and healthcare-system characteristics specific to Taiwan may therefore limit the generalizability of the findings to other populations or settings.

### 4.4. Future Research Directions

Building on the above findings and discussion, several directions may be considered for future research:

1. Examine the relationships among ACEs, MG disease characteristics, and psychological adaptation.
  Future studies should investigate whether ACE exposure is associated with the age of MG onset, symptom severity, or clinical presentation, and how these disease-related factors interact with coping strategies, emotional responses, and illness-related behaviors.

2. Address limitations related to sample size and subgroup distribution.
  The small number of participants in higher ACE categories in the present study may limit statistical stability and increase susceptibility to outlier effects. Future research with larger samples and more balanced subgroup distributions is needed to improve robustness and generalizability of findings.

3. Enhance engagement and participation in online psychological interventions.
  Although online courses provide flexibility and accessibility, participation rates in this study remained relatively low, despite the use of attendance tracking. Future studies may examine strategies to improve adherence and interaction, such as optimizing platform usability, incorporating interactive elements, and employing multi-channel communication approaches. Strengthening these components may increase participation and maximize the potential benefits of digital mental health interventions.

4. Employ randomized controlled designs with longer follow-up periods.
  To better evaluate intervention effects and their persistence over time, future studies should consider randomized controlled trial designs incorporating appropriate comparison conditions (e.g., waitlist or active control groups) and longer follow-up periods. Such designs would allow for a more rigorous evaluation of intervention efficacy and sustainability over time.

## 5. Conclusions

The present study identified age-related patterns of ACEs among patients with MG, with emotional abuse more frequently reported by older adults and physical and sexual abuse more commonly reported by younger adults. Greater ACE exposure was associated with increased depressive symptoms, highlighting the potential long-term psychological impact of early-life adversity in this clinical population. In addition, participation in an online positive mental health BMI learning program was associated with improvements in overall psychological well-being, primarily characterized by enhanced emotional stability. Taken together, these findings suggest that online psychological programs may represent a feasible and potentially supportive approach for addressing psychological well-being in patients with MG. Further controlled studies are warranted to clarify their effectiveness and to better define their role within comprehensive care.

## Figures and Tables

**Table 1:** Sociodemographic Characteristics of Patients with MG (*N* = 77).

Sociodemographic Characteristics	Categories	*N*	%
Gender	Male	18	23.4
	Female	59	76.6
Age	18–44	28	36.4
	45–64	41	53.2
	Over 65	8	10.4
Education	Junior high school and below	3	3.9
	High school	24	31.2
	College or above	50	64.9
Marital status	Single	21	27.3
	Married	45	58.4
	Divorced or widowed	11	14.3
Living arrangement	Living alone	7	9.1
	Live with family	69	89.6
	Other (nursing home)	1	1.3
Club participation	Yes	61	79.2
	No	16	20.8
Employment Status	Full time	42	54.5
	Part-time	5	6.5
	Suspend without pay	1	1.3
	Retire	9	11.7
	Not working	20	26.0
Monthly income	No monthly income	17	22.1
	Low income	19	24.7
	High income	41	53.2

Note: Percentages are calculated based on the total sample (*N* = 77). Low income is NT$30,000 or less per month, and high income is NT$30,000 or more per month. Abbreviation: MG, myasthenia gravis; *N*, patient numbers.

**Table 2:** Endorsement Rates for ACE Questionnaire Among Patients with MG (*N* = 77).

Questions	Options	*N*	Proportion (%)
1. Did a parent or adult in your home ever swear at you, insult you, or put you down? (Item 1 Emotional abuse)	Yes	15	19.5
	No	62	80.5
2. Did a parent or adult in your home ever hit, beat, kick, or physically hurt you in any way? (Item 2 Physical abuse)	Yes	9	11.7
	No	68	88.3
3. Did you experience unwanted sexual contact (such as fondling or oral/anal/vaginal intercourse/penetration)? (Item 3 Sexual abuse)	Yes	7	9.1
	No	70	90.9
4. Did you feel that no one in your family loved you or thought you were special? (Item 4 Emotional neglect)	Yes	11	14.3
	No	66	85.7
5. Did you feel that you didn’t have enough to eat, had to wear dirty clothes, or had no one to protect or take care of you? (Item 5 Physical neglect)	Yes	3	3.9
	No	74	96.1
6. Did you lose a parent through divorce, abandonment, death, or other reason? (Item 6 Parental divorce or separation)	Yes	13	16.9
	No	64	83.1
7. Did your parents or adults in your home ever hit, punch, beat, or threaten to harm each other? (Item 7 Household member treated violently)	Yes	2	2.6
	No	75	97.4
8. Did you live with anyone who had a problem with drinking or using drugs, including prescription drugs? (Item 8 Alcohol and/or drug abuser in the household)	Yes	5	6.5
	No	72	93.5
9. Did you live with anyone who was depressed, mentally ill, or attempted suicide? (Item 9 Someone chronically depressed, mentally ill, institutionalized or suicidal)	Yes	7	9.1
	No	70	90.9
10. Did you live with anyone who went to jail or prison? (Item 10 Incarcerated household member)	Yes	2	2.6
	No	75	97.4

Note: Percentages are calculated based on the total sample (*N* = 77). Abbreviation: ACE, adverse childhood experiences; MG, myasthenia gravis; *N*, patient numbers.

**Table 3:** Associations between ACE Questionnaire Responses and Sociodemographic Characteristics in Patients with MG (ANOVA).

Item	Gender	Age	Education	Marital Status	Living Arrangement	Club Participation	Employment Status	Monthly Income
1 Emotional abuse	0.092	0.020 *	0.288	0.847	0.054	0.935	0.092	0.035 *
2 Physical abuse	0.459	0.024 *	0.230	0.473	0.918	0.911	0.591	0.640
3 Sexual abuse	0.129	0.008 **	0.288	0.501	0.004 **	0.600	0.807	0.064
4 Emotional neglect	0.665	0.274	0.470	0.754	0.048 *	0.173	0.820	0.471
5 Physical neglect	0.682	0.810	0.940	0.567	0.840	0.372	0.681	0.213
6 Parental divorce or separation	0.147	0.220	0.537	0.117	0.083	0.826	0.147	0.168
7 Household member treated violently	0.435	0.856	0.585	0.065	0.000 ***	0.308	0.378	0.317
8 Alcohol and/or drug abuser in the household	0.856	0.723	0.154	0.144	0.671	0.965	0.864	0.715
9 Someone chronically depressed, mentally ill, institutionalized or suicidal	0.738	0.842	0.710	0.997	0.004 **	0.600	0.807	0.845
10 Incarcerated household member	0.435	0.171	0.831	0.492	0.892	0.470	0.802	0.619
Total Score	0.814	0.047 *	0.432	0.929	0.002 **	0.620	0.254	0.191

Note: Values represent *p* values from one-way ANOVA analyses. Analyses were conducted for exploratory purposes and were not adjusted for multiple comparisons. Findings should be interpreted descriptively, particularly for variables with small subgroup sizes. Significance level set at *α* = 0.05, * *p* < 0.05, ** *p* < 0.01, *** *p* < 0.001. Abbreviation: ACE, adverse childhood experiences; MG, myasthenia gravis; ANOVA, analysis of variance.

**Table 4:** Post Hoc Multiple Comparisons of Age Groups for ACE Items Identified in Exploratory Analyses (Items 1–3).

Item	Comparison Methods	Age (I)	Age (J)	Average Difference (I–J)	Standard Error	*p* Value
1 Emotional abuse	Scheffé	18–44	45–64	0.1524	0.0939	0.274
			Over 65	−0.2500	0.1536	0.272
		45–64	18–44	−0.1524	0.0939	0.274
			Over 65	−0.4024	0.1481	0.03 *
		Over 65	18–44	0.2500	0.1536	0.272
			45–64	0.4024	0.1481	0.03 *
2 Physical abuse	LSD	18–44	45–64	0.1899	0.0764	0.015 *
			Over 65	−0.0357	0.1250	0.776
		45–64	18–44	−0.1899	0.0764	0.015 *
			Over 65	−0.2256	0.1205	0.065
		Over 65	18–44	0.0357	0.125	0.776
			45–64	0.2256	0.1205	0.065
3 Sexual abuse	Scheffé	18–44	45–64	0.2143	0.0674	0.009 **
			Over 65	0.0893	0.1102	0.721
		45–64	18–44	−0.2143	0.0674	0.009 **
			Over 65	−0.1250	0.1062	0.504
		Over 65	18–44	−0.0893	0.1102	0.721
			45–64	0.1250	0.1062	0.504

Note: Values represent *p* values from post hoc multiple comparisons conducted for exploratory purposes. Positive values indicate higher ACE endorsement in the first-listed age group compared with the reference group. Significance level set at *α* = 0.05, * *p* < 0.05, ** *p* < 0.01. Abbreviations: ACE, adverse childhood experiences; LSD, least significant difference.

**Table 5:** Post Hoc Multiple Comparisons of Monthly Income Groups for ACE Item 1 (Emotional Abuse) Identified in Exploratory Analyses.

Item	Comparison Method	Monthly Income (I)	Monthly Income (J)	Average Difference (I–J)	Standard Error	*p* Value
Emotional Abuse	Scheffé	None	Low income	0.2539	0.1289	0.151
			High income	0.2898	0.1114	0.039 *
		Low income	None	−0.2539	0.1289	0.151
			High income	0.0359	0.1072	0.945
		High income	None	−0.2898	0.1114	0.039 *
			Low income	−0.0359	0.1072	0.945

Note: Values represent *p* values from post hoc multiple comparisons conducted for exploratory purposes. Positive values indicate higher emotional abuse endorsement in the first-listed income group compared with the reference group. Low income is NT$30,000 or less per month, and high income is NT$30,000 or more per month. Significance level set at *α* = 0.05, * *p* < 0.05. Abbreviations: ACE, adverse childhood experiences.

**Table 6:** Comparison of MG-ADL, MG-QOL15, mBMI, and PHQ-9 Scores Across Pre-test ACE Total Score Groups (ANOVA).

ACE			MG-ADL		MG-QOL15		mBMI		PHQ-9	
Total Score	*N*	%	Average	SD	Average	SD	Average	SD	Average	SD
0	44	57.1	3.59	3.01	12.25	11.52	23.23	5.20	4.84	4.39
1	14	18.2	3.57	3.39	12.43	11.09	23.36	5.37	6.21	4.37
2	8	10.4	3.25	1.58	13.13	12.54	18.88	6.47	7.75	5.06
3	5	6.5	4.40	3.21	18.40	17.56	20.60	4.04	11.80	6.18
4	3	3.9	5.00	4.58	6.67	6.03	24.33	7.37	4.33	4.51
5	1	1.3	8.00	NA	10.00	NA	11.00	NA	10.00	NA
6	2	2.6	4.50	0.71	15.50	13.44	17.50	7.78	15.00	16.97
Total	77	100.0	3.74	2.97	12.61	11.58	22.36	5.63	6.16	5.35
*F*			0.537		0.360		1.965		2.931 *	

Note: Values represent means and standard deviations across ACE total score groups. *p* values are derived from one-way ANOVA analyses. NA indicates values not estimable due to very small subgroup sizes. Significance level set at *α* = 0.05, * *p* < 0.05. Some ACE categories contained very small sample sizes, which may affect the stability and precision of subgroup estimates. Abbreviations: MG-ADL, myasthenia gravis activities of daily living scale; MG-QOL15, myasthenia gravis quality of life 15-item; mBMI, the indicator of mental health BMI on well-being questionnaire; PHQ-9, patient health questionnaire-9; ACE, adverse childhood experiences; ANOVA, analysis of variance; *N*, patient numbers; SD, standard deviation.

**Table 7:** Pre- and Post-test Comparison of MG-ADL, MG-QOL15, mBMI, and PHQ-9 scores After the Online Positive Mental Health BMI Learning Program.

		*N*	Mean	SD	Sample Correlation	*T*	*p* Value
MG-ADL	Pre-test total score	32	4.00	2.98	0.421	0.976	0.337
	Post-test total score	32	3.47	2.86			
MG-QOL15	Pre-test total score	32	15.66	13.26	0.453	0.718	0.478
	Post-test total score	32	14.06	10.24			
mBMI	Pre-test total score	32	21.41	4.70	0.763	−2.900	0.007 **
	Post-test total score	32	23.03	4.49			
PHQ-9	Pre-test total score	32	6.25	4.40	0.637	0.476	0.638

Note: Analyses were conducted among participants who completed both pre- and post-test assessments. Significance level set at *α* = 0.05, ** *p* < 0.01. Abbreviations: MG-ADL, myasthenia gravis activities of daily living scale; MG-QOL15, myasthenia gravis quality of life 15-item; mBMI, the indicator of mental health BMI on well-being questionnaire; PHQ-9, patient health questionnaire-9; *N*, patient numbers; SD, standard deviation.

**Table 8:** Pre–Post-test Comparison in mBMI Subscales Following the Online Positive Mental Health BMI Program.

mBMI		*N*	Mean	SD	Sample Correlation	*T*	*p* Value
1. I can find good friends to support and accompany me when I need them. How confident am I on a scale of 0 to 10? (B)	Pre-test total score	32	6.88	2.23	0.759	−1.740	0.091
	Post-test total score	32	7.34	2.16			
2. I can detect the changes in my inner emotions and keep myself calm. How confident am I on a scale of 0 to 10? (M)	Pre-test total score	32	7.25	1.68	0.604	−2.420	0.022 *
	Post-test total score	32	7.84	1.37			
3. I can recognize my own importance and the value of life. How confident am I on a scale of 0 to 10? (I)	Pre-test total score	32	7.28	2.16	0.655	−1.890	0.069
	Post-test total score	32	7.84	1.83			

Note: Analyses were conducted among participants who completed both pre- and post-test assessments. Significance level set at *α* = 0.05, * *p* < 0.05. Abbreviations: mBMI, the indicator of mental health BMI on well-being questionnaire; *N*, patient numbers; SD, standard deviation.

## Data Availability

The data presented in this study are available on request from the corresponding author.

## Abbreviations

The following abbreviations are used in this manuscript:

ACE	Adverse childhood experience
ANOVA	Analysis of variance
CDC	Centers for Disease Control and Prevention
DSM-IV	Diagnostic and Statistical Manual of Mental Disorders-IV
LSD	Least significant difference test
mBMI	The indicator of mental health BMI on well-being
MG	Myasthenia gravis
MG-ADL	Myasthenia Gravis Activities of Daily Living Scale
MG-QOL	Myasthenia Gravis Quality of Life
MG-QOL15	Myasthenia Gravis Quality of Life 15-item
*N*	Patient numbers
PHQ-9	Patient Health Questionnaire-9
QMG	Quantitative Myasthenia Gravis
SPSS	Statistical Product and Service Solutions
SD	Standard deviation
